# Cow’s milk allergens as an infrequent cause of anaphylaxis to systemic corticosteroids

**DOI:** 10.1186/2045-7022-1-S1-P18

**Published:** 2011-08-12

**Authors:** Savvas Savvatianos, Staxroula Giavi, Evangelina Stefanaki, George Siragakis, Emmanouil Manousakis, Nikolaos G Papadopoulos

**Affiliations:** 1 University of Athens, 2nd Pediatric Clinic, Allergy Dept, Athens, Greece

## Background

Immediate allergic reactions to corticosteroids are uncommon, while causative agents usually involve the steroid molecule or an excipient. Here we report 2 cases of acute reaction to methyl-prednisolone (MP), attributed to milk allergen contamination.

## Case 1

A 9-year-old boy with a history of severe persistent cow’s milk allergy (CMA) was seen at the Emergency Department due to a virus-induced asthma exacerbation presented with wheezing and moderate dyspnea. The boy was administered nebulized salbutamol and 40mg of MP intravenously. Wheezing deteriorated, so the boy was given another course of the same medication. Within a few minutes the patient acutely collapsed, with hypotension, cyanosis and respiratory arrest and had to be immediately transferred to the IC Unit, where he was injected epinephrine and was intubated.

## Case 2

A 7-year-old boy with severe CMA was similarly treated with salbutamol and intravenous administration of 40mg MP, following clinical diagnosis of an asthma exacerbation. The therapeutic intervention resulted in a full-blown anaphylactic reaction, with aggravation of dyspnea and wheezing, emesis, rash and hypotension.

## Diagnostic workout

Both children were evaluated within 6 months for assumed IgE-mediated reactivity to MP. Skin-testing was performed using 7 different corticosteroids to evaluate cross-reactivity pattern and identify the culprit agent. Both patients only produced positive results to lactose-containing preparations. Subsequent drug provocation test was negative in both patients for a full therapeutic dose (125mg) of non-lactose containing MP preparation. By employing a high-sensitivity ELISA assay, we managed to detect *traces of casein and β-lactoglobulin* in all samples from 6 batches of the implicated product (Solu-Medrol 40mg, Pfizer), proving our hypothesis of milk allergen contamination.

## Conclusion

Pharmaceutical lactose, contained as an excipient in corticosteroid preparations may be an iatrogenic cause of anaphylaxis in children with severe CMA, due to milk protein contamination. Non-lactose containing preparations should be exclusively used when treating such patients.

